# The Predictors of Psychological Well-Being in Lithuanian Adolescents after the Second Prolonged Lockdown Due to COVID-19 Pandemic

**DOI:** 10.3390/ijerph19063360

**Published:** 2022-03-12

**Authors:** Roma Jusienė, Rima Breidokienė, Stanislav Sabaliauskas, Brigita Mieziene, Arunas Emeljanovas

**Affiliations:** 1 Faculty of Philosophy, Institute of Psychology, Vilnius University, LT-01513 Vilnius, Lithuania; rima.breidokiene@fsf.vu.lt; 2 Faculty of Medicine, Institute of Health Sciences, Vilnius University, LT-03101 Vilnius, Lithuania; stanislav.sabaliauskas@mf.vu.lt (S.S.); brigita.mieziene@mf.vu.lt (B.M.); arunas.emeljanovas@mf.vu.lt (A.E.)

**Keywords:** adolescents, psychological well-being, COVID-19, physical activity, social support

## Abstract

Recent research highlights the impact of prolonged pandemics and lockdown on the mental health of youngsters. The second wave of COVID-19 brought an increase in mental health problems among young people. Therefore, this study aims to analyze the main factors arising from intra-individual, inter-individual, and environmental contexts that predict good psychological well-being in a group of adolescents after a second prolonged period of social restrictions and distance education. The study included 1483 school students from 11 to 19 years old. The survey assessed self-reported students’ psychological well-being (WHO-5 index), physical activity, sedentary behavior, school social capital, communication with peers and relationships with parents, existing emotional and behavioral problems. The results indicated that 58% of adolescents were of good psychological well-being in spring 2021, after half a year in lockdown. Almost 19% of adolescents had depression risk. The study revealed that during a period of prolonged isolation, male gender, better relationships between young people and their parents, the absence of serious emotional and behavioral problems, less sedentary behavior, and higher school social capital were found to be significant factors predicting adolescents’ psychological well-being. Lower physical activity is an important contributor to students’ poor well-being. Finally, the lack of face-to-face communication with peers was revealed as a specific factor in predicting adolescents with depression risk.

## 1. Introduction

The pandemic due to the unpredictable and high spread of the coronavirus SARS-CoV-2 resulted in an infectious disease called COVID-19 that affected the lives of billions of people over the world, both directly and indirectly—through restrictions and major changes in everyday lives. From the biopsychosocial perspective, the major challenge was to balance between controlling the spread of the virus, staying healthy in terms of the infectious disease, and meeting the essential psychosocial needs. Although the negative impact of the lifestyle changes, stay-at-home orders, lockdowns, and therefore social restrictions, was documented for various groups of the population, the adolescents might be especially vulnerable because of the developmental tasks and challenges [1,2,3,4]. Moreover, school-aged children might be experiencing additional specific strains because of prolonged school closure and distance education, and therefore reduced professional support, reduced social activities, and increased physical isolation from peers [3,5,6,7,8,9]. Thus, in adolescence, the additional pandemic challenges might have complicated every aspect of developmental strains and challenges posed by rapid physical and emotional growth itself, including increasing academic demands and expectations, changing social relationships with family and peers, and increasing exposure to online interactions [10,11].

Plenty of studies researching the immediate impact of the pandemic on adolescents’ mental health provided the following clear evidence that: (1) it deteriorated remarkably, especially through an increase in depressive and anxiety symptoms, and self-injury behavior [3,4,12]; (2) physical activity, access to recreational activities, positive family relationships and social support worked as significant protective factors [4,13]. There are only a few studies to date on the impact of the prolonged effects of pandemic/lockdown on the mental health in youngsters [3,14,15], and these studies indicate the higher incidence of mental health problems during the second wave (and therefore lockdown) comparing to the first wave and/or to the pre-pandemic.

Psychological well-being is an important indicator of mental health [16]. In addition, it is important to study factors that are associated with better psychological well-being, especially in times of crisis and particularly among children and youth who are vulnerable to the changes the crisis brings in. Physical activity (PA) is the most researched predictor of psychological well-being during the pre-pandemic and pandemic periods. According to many studies, physical activity is associated with better psychological health in general [17,18] and in times of crisis [19,20]; however, children’s and adolescents’ PA, while being low enough pre-pandemic [11,17,21,22], has significantly decreased [4,13,20,23,24].

Similarly, recreational screen time and overall sedentary behavior had significantly increased during the lockdown due to COVID-19 [13,23,25], and it was associated with more psychosomatic complaints, more depressive symptoms, and lower life satisfaction both in pandemic [13,26] and pre-pandemic [17].

Although adolescents’ relationships with parents seemed to be least impacted in a pandemic, the more favorable relations and more support in the family were also related to mental well-being [4,12]. Moreover, it worked as an important factor mediating the limitations of social support and lack of social interactions with peers [12]. On the other hand, increased conflict with parents and parental distress predicted more emotional and behavioral problems in children and adolescents [3,25,27]. For children living in vulnerable environments, schools can act as a “protective layer” by providing mental health support and alternative social experience [7,28]. As schools play a critical role in children’s social development and are an integral part of the social support system in society [6], adolescents’ subjective evaluation of school as a social resource could be very important [22]. School social capital represents school students’ psychosocial resources such as social support, trust, reciprocity, social norms, social participation, integrity, and cohesion [29] available at school to facilitate educational outcomes and also to attain other life goals. School social capital refers to investments between students and schools, social bonds, and relationships that students form with school teachers and personnel [30]. The question is whether and how the school social capital is related to psychological well-being in distance education when youngsters learn at home.

In researching the psychological well-being in a period of adolescence, the connectedness to peers and friendships deserves special attention. As schools have been closed, extracurricular and leisure activities have been canceled, peer socializing has been severely limited, and adolescents have turned to social media and online communication [10]. However, have the online connections successfully substituted the in-person or face-to-face communication? Importantly, Ellis, Dumas, and Forbes (2020) revealed that more time connecting to friends virtually during the pandemic was related to greater depression in adolescents, but not related to loneliness [12]. Halldorsdottir et al. (2021) have also found passive social media use was related to higher depressive symptoms among adolescent girls [26].

Finally, the individual factors such as female gender and neurodevelopmental adversities and/or preexisting health problems were quite unambiguously related to lower psychological well-being during pandemic and pre-pandemic. The female gender was found to be associated not only with mental health problems, but also more time spent sedentarily and less physical activity [2,11,17,19,26,31,32]. Preexisting health problems [1] and vulnerability due to previous and continuous emotional problems pose additional challenges for the youngsters, especially if the availability of support at schools and at healthcare systems is restricted [5,7]. Several pre-pandemic studies also revealed that older adolescents report more mental health problems [11] and less physical activity [19,22], although studies examining adolescents’ well-being during pandemics did not find age differences [3].

The described above deterioration of school students’ psychological well-being along with the decreased physical activity during the pandemic again indirectly confirms the evidence on their close relationship determined by previous studies [17,33]. In turn, it seems that mental health and physical activity have a common denominator, i.e., social ties and support that comes from social relationships. Recent studies demonstrated that the trust and support which come from school are of the utmost importance for students’ health behaviors, keeping students motivated to be physically active out of school [22], and along with higher physical activity increase the probability of higher self-rated health among school students [34]. This leads to the premise that regular contact and meaningful relationships in school settings are crucial for school students to stay physically and mentally healthy, and that the damage in the social arena of students’ life leads to poor outcomes in other areas such as health behaviors and mental health.

In Lithuania, the first national lockdown (also school closure and switch to home learning) was announced on 16 March 2020 and proceeded till 16 June 2020. After the first lockdown, a significant but mild increase in mental health problems was observed [25,35]. To note, the spring and summer period in this Baltic country is favorable in terms of going outside for sports, walking, other activities, however, no educational and school activities were organized till the summer holidays (which, in Lithuania, last from the middle of June to 1 September). From the beginning of November 2020, the second national lockdown was introduced, which started with the closing of schools and ending of extracurricular activities, and then moved education to youngsters’ homes. Secondary school students (aged 11–19 years) in distance learning have spent more than half a year (from October or November 2020 till May or June 2021), the majority of them returned to regular education at schools only in September 2021. Some students with special education needs and/or from families experiencing socio-economic adversities were allowed to have some contact education or to go to the school building during the distance education period from the end of January 2021. Additionally, a small selection of children and adolescents (e.g., those with high sports achievements) were allowed to have their sports activities in contact from the end of January 2021. Moreover, the important COVID-19 related restriction imposed by the Lithuanian government was that households were not allowed to meet indoors with another household (family). Thus, adolescents’ meetings with friends were also almost impossible, except for meeting one friend outdoors. “Social bubbles” (e.g., meeting and keeping safe contacts with one-to-two families and/or persons who live separately) were allowed only for lonely elders and for single-parent families with young children.

First of all, it is of utmost importance to study whether and how the prolonged school closures, strict social distancing measures, and the pandemic itself affected the well-being of children and adolescents [7]. The adolescents might face many specific pandemic-related risks, e.g., higher sedentary behavior, physical inactivity, and less face-to-face contact. Moreover, the pre-existing emotional and behavioral problems and, probably, female gender might act as additional risk factors in predicting adolescents’ psychological well-being. On the other hand, family support (e.g., satisfactory relationships with parents) and social support at school (e.g., school social capital, even if in distance education) could act as protective factors. There is still a lack of studies analyzing the combination of risk and protective factors stemming from the various contexts, e.g., intra-individual, inter-individual, environmental, and policy [23], during the pandemic. Thus, the current study aims to analyze the predictive value of the main above-mentioned factors, for psychological well-being in a large Lithuanian population-based sample of adolescents after the second prolonged lockdown and distance education.

## 2. Materials and Methods

### 2.1. Participants

The study included 1483 school students across 5th to 12th grade from 11 to 19 years old. The average age was 14.62 (2.03) years. Among them, 56.9% were girls, and 50% were in grades 5–9th.

### 2.2. Study Design and Procedure

This is a cross-sectional population-based study based on a cluster (area) random sampling. The study sample was selected across all 10 regions of Lithuania from May to June of 2021. At that point, the participants were in distance education, which had been introduced in November 2020 and lasted till June 2021. In total, 33 schools participated in the study. Two-thirds (67.7%) of participants represented region centers, and 32.3% represented rural areas in the county. In each selected school, one class per 5th to 12th grade was provided study e-questionnaires. Both the school and classes in the schools were considered clusters. The filling questionnaire took approximately 30 min. The study was conducted following the Declaration of Helsinki, and the protocol was approved by the Ethics Committee of Psychological Research of Vilnius University (No. 66).

Researchers obtained the permission of the school administration to collect data. Links to the online questionnaires for students and informed consent for parents were provided by the researchers and sent by the school administration. Informed consent was collected from the parents online. Students whose parents did not give consent, or who themselves refused to participate, did not participate in the study.

### 2.3. Measures

Psychological Well-being was assessed using the WHO-5. It is a short questionnaire consisting of 5 questions indicating the frequency of being active, vigorous, rested, relaxed, having interests, and being in good spirits, with the answers on the Likert scale from 0—“none of the time” to 5—“all of the time”. The scale has adequate validity both as a screening tool for depression and as an outcome measure in clinical trials, and it has been applied successfully as a generic scale for well-being across a wide range of study fields [36]. The WHO-5 has been translated into over 30 languages and has been used in research projects all over the world [36]. The Lithuanian version of WHO-5 has been used in the HBSC study (Health Behaviour in School-Aged Children, World Health Organization collaborative cross-national survey) with students aged 11 to 15 years old [32]. Scale reliability assessed with Cronbach alpha for this study sample was 0.907. For the statistical analysis, the WHO-5 index score and also the categorical variable based on the cut-off of the index were used. The index score is the sum of 5 items multiplied by 4 (ranges from 0 to 100). Good well-being is considered when the index falls into a range from 51 to 100; poor well-being is considered when the index falls into the range from 29 to 50, and the risk for depression is considered when the index is 28 and less (prof. K. Šmigelskas, the leader of HBSC study group in Lithuania, personal communication).

Physical activity was measured with a question: “How much time per day on average do you spend doing sports and/or exercises in such a way you sweat and increase your breath?”. Students had to select one of the following answers: “not at all”; “up to 30 min”; “31 up to 60 min”; “61 min and more”. Sufficient PA was considered if adolescents indicated doing exercise for at least 61 min daily [37]. The dichotomous variable was coded as 0, which indicated a non-sufficient PA, and 1—a sufficient PA.

Sedentary behavior was assessed using the following question: “How much time per day on average do you spend while sitting (please, count the time you spend at home for learning and leisure, sitting by the table, screen, reading, etc., do not count time for sleeping).” Participants had to provide average duration in hours. Thus, the higher score indicates a more sedentary behavior.

Relationships with parents were assessed with a question: “Please rate your relationships with parents from 1 (bad or very bad) to 5 (good or very good)”. Thus, the higher score indicates more favorable relationships.

School social capital was measured with the School Social Capital Scale consisting of five items, which was developed based on the previous study among Lithuanian school-aged children [22]. The scale represents general school trust, vertical trust (trust in teachers), horizontal trust (trust in school-mates), communication at school, reciprocity (e.g., “Do you think students collaborate in your high school?”). The mean score of five items was used in this study. Scale reliability in the current study measured with Cronbach α is 0.879.

Communication with peers was identified by two separate items, indicating live (face-to-face) and online communication with peers (e.g., “Please identify how often you interact (live or online) with your classmates/peers outside of class this semester”). Answers for the communication were from 1—“Never” to 5—“Several times a week and more”, thus a higher score indicates the more frequent online or face-to-face communication with peers.

Preexisting emotional and behavioral problems were derived from answers to the following two questions: “Overall, do you think that you have difficulties in one or more of the following areas: emotions, concentration, behavior or being able to get on with other people?” (“No”; “Yes, minor difficulties”; “Yes, definite difficulties”; “Yes, severe difficulties”) and “If you have answered “Yes”, please answer, how long have these difficulties been present?” (“Less than a month”; “during this half a year”; “more than half a year up to one year”; “more than a year”). These questions come from the Impact Scale of the Strengths and Difficulties Questionnaire [38]. In the present study, the variable was coded as “no problems or problems with a recent onset”—0; “preexisting emotional and behavioral problems”—1, if a participant indicated definite or severe difficulties and if these difficulties have been present for more than a year.

Sociodemographic variables gender (1—“Girl”, 2—“Boy”) and age in full years were also assessed.

### 2.4. Statistical Analysis

Data were analyzed using SPSS 24.0 (SPSS Inc., Chicago, IL, USA) software. The distribution of variables in the groups was calculated using frequency distribution tests. Relationships between the variables were calculated using Spearman’s correlations. Comparisons of means between groups were conducted with the Kruskal–Wallis test (3 groups), and the Chi-squared test was used to evaluate differences between categorical or dichotomous variables. Multinomial logistic regression was used to predict categorical placement in or the probability of category membership on a dependent variable well-being index based on multiple independent variables. The dependent variable (WHO-5 well-being index) was transformed into three categories and coded as follows: 1—“good” 2—“poor”, 3—“depression risk”. STROBE Statement-checklist guidelines were followed in organizing this paper.

## 3. Results

The baseline characteristics of the sample are presented in Table 1. The results indicated that about one-fourth of the sample evaluated their psychological well-being as poor and 18.6% had a depression risk in late spring of the year 2021. Only 23.1% of adolescents were sufficiently physically active during this period. The average duration of the participants’ sedentary behavior was more than 8 h per day. Nearly 9% of the participants had definite or severe behavioral and emotional problems, lasting more than a year. Only one-third of the adolescents had face-to-face communication with their peers several times per week or more, and almost one-fifth of the participants had no face-to-face communication with peers over the past 6 months. In comparison, almost 60% of adolescents had frequent (several times per week and more) online communication with their peers.

Girls had lower scores of WHO-5 index (F = 3.63, df = 1481, *p* < 0.001). Girls also rated their school social capital (F = 0.29, df = 1481, *p* < 0.001) and relationships with parents as poorer (F = 5.85, df = 1437.96, *p* = 0.25), but sedentary behavior duration as longer (F = 0.07, df = 1481, *p* < 0.001) in comparison with boys. Boys were more physically active (χ^2^ = 40.66, df = 3, *p* < 0.001), but had less face-to-face communication with peers (χ^2^ = 9.06, df = 3, *p* = 0.028) in comparison with girls. More girls than boys reported having emotional and behavioral problems lasting more than a year (χ^2^ = 11.48, df = 3, *p* = 0.001). No gender differences were found in online communication with peers.

Further, we performed the correlational analysis of the study variables (presented in Table 2). Poorer well-being was related to older age, more sedentary behavior, less physical activity, worse-evaluated relationships with parents, less frequent face-to-face and online contacts with peers, and lower scores of school social capital. Elder children were less physically active and were more engaged in sedentary behavior, but they had more face-to-face and online contact with peers and better assessed their school social capital. The more sedentary behavior was correlated with less physical activity, less frequent face-to-face contact with peers, worse relationships with parents, and lower scores of school social capital. Adolescents who were more satisfied with their relationships with parents had more face-to-face and online interactions with their peers and rated their school social capital as higher.

In the final stage of analysis, a multinomial logistic regression was performed for the mental well-being index as a categorical outcome variable. In the multivariate analysis, we included the following independent variables: child age and gender, sedentary behavior duration and physical activity, relationships with parents, frequency of face-to-face communication with peers, frequency of online communication with peers, school social capital, and the preexisting emotional and behavioral problems. The results presented in Table 3 show that male gender, less sedentary behavior, no mental health problems over the last year, better relationships with parents and higher school social capital, as well as more frequent face-to-face interactions with peers increased the probability of good well-being of adolescents as opposed to those who had a depression risk. Male gender, later onset of problems, better relationships with parents, higher social capital, and more frequent face-to-face communication with peers increased the probability of poor well-being versus depression risk. Finally, male gender, less sedentary behavior, more physical activity, no mental health problems over the last year, better relationships with parents, and higher scores of school social capital predicted good well-being versus poor well-being (see Table 3).

## 4. Discussion

The pandemic has fundamentally altered the way of everyday living in various age groups, although adolescents’ lifestyle and therefore psychological well-being might be specifically affected. First, during the pandemic, they spent prolonged periods conducting distance learning and had to adhere to considerable restrictions of social activities and contact connections with peers. Secondly, this age period is marked with additional developmental tasks and challenges, mostly related to self-identity through socializing and formal and informal activities at schools and public places. Thus, it is very important to reveal and/or confirm the main factors which help adolescents stay in good psychological well-being during the prolonged periods of pandemic-related strains and challenges. In addition, it is important to analyze the risks which predict the probability of deterioration of psychological well-being and/or of becoming depressed.

More than half (58%) of the adolescents aged 11 to 19 years old in a Lithuanian population-based sample of this study self-evaluated their psychological well-being as good after half a year of the second lockdown and distance learning in the period April–June 2021. To note, 69% of adolescents have been evaluated as having good psychological well-being using the same methods in April–June 2018 [32]. Nearly one-fifth of adolescents (18.6%) in our study were considered as having a depression risk when assessed with the WHO-5. To compare, 12.1% of Lithuanian adolescents aged 12 to 16 years old had any mental disorder, and only 2.4% had a diagnosis for depression in an epidemiological study conducted in 2004–2007 [39]. Therefore, the results of our study confirm the increase in mental health problems in adolescents during the pandemic and add to the similar results of lots of recent studies over the various countries [4], also indicating the worsening of the mental health in children and adolescents during the second wave of pandemic restrictions [14,15].

In the present study, better relationships with parents, male gender, absence of definite or severe emotional and behavioral problems during the past year, lower sedentary behavior, and higher school social capital were revealed as significant factors in differentiating adolescents with good well-being from adolescents with poor well-being and depression risk. Physical activity was found to be an additional factor to differentiate good well-being adolescents from poor well-being in adolescents. Finally, the lack of face-to-face communication with peers was revealed as a specific factor in predicting adolescents with depression risk (as compared to those with good well-being and poor well-being). The results are supported by previous studies in a similar population of adolescents. For instance, back in pre-pandemic period, it was found that male high school students, having low distress, being physically active more days per week, as well as perceiving high trust in teachers, trust in peers, and reciprocity at school, rated their health above average [34]. The association of self-rated health in adolescents is especially strongly related to psychological well-being [40] as adolescents address their health ratings to their psychological domain, while their physical health usually is not yet deteriorated. Regarding the pre-existing emotional problems and their relationship with lower psychological well-being in the current study, it might be explained by biological dysregulation of the stress process when previous exposure to stressors induce vulnerability to subsequent exposure, which can lead to failure to develop an adaptive response in the face of subsequent trauma (such as pandemic-related outcomes) and increase the risk for the deepening of already existing psychological problems [41]. Results indicate that school students with increased emotional vulnerability, i.e., previously exposed to psychological traumas, need additional attention to their psychological health and help in building capacity for resilience in a pandemic-like or another kind of crisis.

It was found that adolescent girls had lower well-being and a higher risk of depression during the pandemic. These results are in line with the most recent studies [2,3,12,26] as well as with most of the pre-pandemic large-scale epidemiological studies [11,39]. Moreover, female youngsters in our study were less physically active and reported a longer duration of sedentary behavior, which was also prominent in other studies showing that girls in adolescence usually exhibited less healthy lifestyle also during pre-pandemic times [11,22]. Thus, healthcare professionals should pay specific attention to female teenagers’ health-related behaviors and provide them with additional support during challenging times. Additionally, it would be worth further analyzing if adolescent girls and boys could be differentially affected by various risk factors [26].

Higher physical activity and less sedentary behavior were the significant predictors of good well-being in adolescence, supporting the existing evidence-based results suggesting that these are very important for keeping youth in a good health and good mood [23], especially in high-income countries [17]. The current study, as well as other studies, reports high proportions of the adolescents not being sufficiently physically active and/or showing an excessive duration of passive screen time during the pandemic [13,19,20,42] and pre-pandemic [10,11,17,21,22,23,32] periods. These findings are particularly concerning. The proactive and intense actions of schools and/or public healthcare providers to implement and steadily sustain the programs for motivating physical activity in adolescents are highly encouraged. To note, Lithuania and several other Nordic countries are regions with unfavorable conditions to have sports activities outdoors in autumn, winter, and early spring of 2021, the periods which were restricted for any indoor sports activities for most adolescents during the second lockdown. Therefore, it should be ensured that children and adolescents still have access to sport and exercise during possible future periods of closure [19], for example, by maintaining sports clubs and sports facilities that comply with the existing COVID-19 rules [20]. Our previous studies also revealed that after controlling for sociodemographic factors, youths’ leisure physical activity was related to higher accessibility to physical activity resources, neighborhood safety, family social capital, and to the greater social network and social participation, implying that the interventions should include a community’s social and physical environmental changes [22]. Finally, public health strategies to promote adolescents’ mental well-being should aim to increase physical activity and decrease sedentary screen time simultaneously [17]. Higher sedentary behavior is related to lower PA, although these two are not overlapping [25]. In the present study, the shorter duration of sedentary behavior predicted good well-being together with (not interchangeably) sufficient PA.

The results of our study also add to the well-established literature highlighting social support as an important factor of psychological well-being during crisis and stressful life events. Better relationships with parents, together with higher school social capital (e.g., revealing higher trust, reciprocity, and connectedness to teachers and classmates) and more frequent communication with peers, were all significant predictors of good well-being. Based on the results of the current study, we claim that for good psychological well-being in adolescence, the social relatedness and support coming from various contexts (e.g., family, school, friendships) do not necessarily substitute each other during the prolonged challenging periods. To note, the school social capital reflecting the adolescents’ sense of belonging to the school community was also important, keeping in mind the fact that youngsters were in distance learning for more than half a year. Thus, school authorities and teachers should aim to maintain students’ social connectedness to the school community, despite the physical isolation.

Importantly, online communication with peers was not significant in the prediction of psychological well-being. On the contrary, the lack of communication between peers was the significant predictor of the adolescents who had a risk of depression, as opposed to those who had good or less favorable well-being. Thus, first, these results showed that being socially disconnected during the pandemic was related to more depressive moods or lower psychological well-being, also proposed by other researchers [3,4,8,43]. Secondly, our results brought important arguments to the initial discussion on whether social media and online communication with friends can effectively satisfy the social needs of adolescents during the school closure and overall social restrictions [10,12]. The results of our study propose that in-person communication with classmates/peers could not be simply displaced with online communication. Social media use for connectedness can be paralleled with the intake of low-nutrition calorie-dense food for satisfaction of physiological needs. This food could help to satisfy hunger or to survive when no other food is available in the short term. However, if it is consumed without healthy food and for a long time, it will necessarily lead to serious health problems. Finally, keeping in mind that adolescence is also an important period to form and evolve romantic relationships, further studies should also bring more evidence-based clarity on whether and how the restrictions of face-to-face contacts and displacement with technology-based communication may affect romantic development and intimacy [10].

Last but not least, there are obvious differences in the level of psychological well-being of adolescents across countries [2], mostly because of variations in socioeconomics and policy decisions regarding the pandemic measures applied (e.g., the longevity and format of the restrictions). In addition, the particular seasonal and geographical differences might also imply various outcomes of the same restrictions implied, e.g., youths in the northern zone of Europe might be experiencing considerable difficulties in meeting friends outdoors in autumn and winter, while the social activities and in contact meetings indoors were heavily restricted by local policies. Thus, while implementing the necessary national restrictions to prevent the spread of the virus, policymakers should also carefully evaluate the additional country-specific hazards for the well-being of children and adolescents to meet their essential psychosocial needs.

This is a large-scale population-based research study that also covered the analysis of several important intra-individual, inter-relational and environmental factors. Nevertheless, it has several methodological limitations. First of all, it is a cross-sectional study, thus it does not provide evidence on the causality of effects. In addition, the self-reporting measures used in the study could be biased toward participant adolescents’ moods. Finally, data were collected within a single country (Lithuania), thus the results of it might not be generalizable to the other countries.

Despite these limitations, this research contributes to the many related studies by revealing the significant factors which are important in maintaining the youngsters’ psychological well-being during the prolonged lockdown due to COVID-19. The study showed that adolescents with sufficient physical activity, lower sedentary behavior, better relations with parents, schools, and peers, as well as having the possibility to meet peers in-person, stayed in good psychological well-being after half a year in a national lockdown and distance education. In line with a recent systemic review [4] we emphasize the need for practitioners and policymakers to pay more attention to children and adolescents, especially those at high risk, to mitigate the short- and long-term effects of the pandemic on mental health of children and youth. While the pandemic is not yet controlled and the social restrictions are on the way, governments should provide the families and communities with the specific measures which help to monitor adolescents’ psychological well-being, to safely meet their social needs, and to further develop their resilience in coping with crises.

## Figures and Tables

**Table 1:** Baseline characteristics of the participants.

Characteristics	(*n* = 1483)
Child age (years) ^a^	14.62 (*SD* = 2.03)
Child gender	
% Girls	56.9 (*n* = 844)
% Boys	43.1 (*n* = 639)
WHO-5 well-being index ^a^	54.63 (*SD* = 24.20)
% Good well-being	58.3 (*n* = 865)
% Poor well-being	23.1 (*n* = 342)
% Depression risk	18.6 (*n* = 276)
Sedentary behavior (h) ^a^	8.60 (*SD* = 3.28)
Physical activity	
% Not-Sufficient	76.6 (*n* = 1136)
% Sufficient	23.4 (*n* = 347)
Preexisting emotional and behavioral problems	
% No problems or within the past year	91.5 (*n* = 1351)
% More than a year ago	8.5 (*n* = 125)
Relationships with parents ^a^	4.00 (*SD* = 0.97)
Face-to-face communication with peers	
% Never	19.4 (*n* = 288)
% Several times per half-a-year	23.5 (*n* = 348)
% Several times per month	25.7 (*n* = 381)
% Several times per week and more often	31.4 (*n* = 466)
Online communication with peers	
% Never	9.2 (*n* = 13)
% Several times per half-a-year	11.33 (*n* = 168)
% Several times per month	19.1 (*n* = 283)
% Several times per week and more often	60.4 (*n* = 895)
School social capital ^a^	3.65 (SD = 0.79)

^a^ Values are presented in means and standard deviations (*SD*).

**Table 2:** Bivariate correlations among study variables.

Variable	1	2	3	4	5	6	7
1. Well-being index	-						
2. Age	−0.09 **	-					
3. Sedentary behavior	−0.26 ***	0.15 ***	-				
4. Physical activity	0.24 ***	−0.09 **	−0.25 **	-			
5. Relationships with parents	0.35 ***	−0.07 *	−0.13 ***	0.14 ***	-		
6. Face-to-face communication with peers	0.17 ***	0.08 **	−0.10 ***	0.12 ***	0.12 ***	-	
7. Online communication with peers	0.08 **	0.07 *	−0.01	0.01	0.12 ***	0.28 ***	-
8. School social capital	0.36 ***	0.08 **	−0.08 **	0.08 **	0.32 ***	0.23 ***	0.22 ***

* *p* < 0.05, ** *p* < 0.01, *** *p* < 0.001.

**Table 3:** Multinomial regression analysis representing the independent predictors of well-being (WHO-5).

	Well-Being Index (WHO-5)					
	Good Well-Being vs. Depression Risk		Poor Well-Being vs. Depression Risk		Good Well-Being vs. Poor Well-Being	
Variables	OR (95% CI)	*p*	OR (95% CI)	*p*	OR (95% CI)	*p*
Age	0.98 (0.91–1.07)	0.679	1.04 (0.96–1.13)	0.363	0.95 (0.88–1.01)	0.104
Gender (ref.—male)	0.36 (0.25–0.50)	<0.001	0.59 (0.40–0.85)	0.005	0.61 (0.46–0.80)	<0.001
Sedentary behavior	0.90 (0.86–0.95)	<0.001	0.96 (0.91–1.01)	0.126	0.94 (0.90–0.98)	0.003
Physical activity (ref.—sufficient)	0.68 (0.44–1.03)	0.069	0.98 (0.61–1.55)	0.917	0.69 (0.49–0.98)	0.035
Preexisting emotional and behavioral problems (ref.—more than a year age)	5.54 (3.15–9.76)	<0.001	1.80 (1.12–2.89)	0.014	3.08 (1.74–5.45)	<0.001
Relationships with parents	1.80 (1.52–2.12)	<0.001	1.29 (1.09–1.53)	0.003	1.39 (1.19–1.62)	<0.001
Face-to-face communication with peers	1.28 (1.10–1.49)	0.001	1.22 (1.04–1.44)	0.016	1.05 (0.92–1.20)	0.459
Online communication with peers	1.08 (0.92–1.27)	0.327	1.08 (0.91–1.29)	0.361	1.00 (0.87–1.16)	0.992
School social capital	1.88 (1.51–2.34)	<0.001	1.18 (0.94–1.48)	0.150	1.59 (1.31–1.92)	<0.001

OR—odds ratio, 95% CI—95% confidence interval.

## Data Availability

The data presented in this study are available on request from the corresponding author.
